# Feature stability and setup minimization for EEG-EMG-enabled monitoring systems

**DOI:** 10.1186/s13634-022-00939-3

**Published:** 2022-10-27

**Authors:** Giulia Cisotto, Martina Capuzzo, Anna Valeria Guglielmi, Andrea Zanella

**Affiliations:** 1 Department of Information Engineering, University of Padova, Via Gradenigo, 6, 35121 Padova, Italy; 2 Inter-University Consortium for Telecommunications (CNIT), Padova, Italy; 3 Department of Informatics, Systems and Communications, University of Milano-Bicocca, Viale Sarca, 336, 20126 Milano, Italy; 4 Human Inspired Technologies Research Center, University of Padova, Via Luzzatti, 4, 35121 Padova, Italy

**Keywords:** Consensus clustering, Cyber-physical systems, Data fusion, EEG, EMG, Feature selection, Feature stability, Human activity recognition, Magnitude-square coherence, Mobile health, Multi-modal, Usability

## Abstract

Delivering health care at home emerged as a key advancement to reduce healthcare costs and infection risks, as during the SARS-Cov2 pandemic. In particular, in motor training applications, wearable and portable devices can be employed for movement recognition and monitoring of the associated brain signals. This is one of the contexts where it is essential to minimize the monitoring setup and the amount of data to collect, process, and share. In this paper, we address this challenge for a monitoring system that includes high-dimensional EEG and EMG data for the classification of a specific type of hand movement. We fuse EEG and EMG into the magnitude squared coherence (MSC) signal, from which we extracted features using different algorithms (one from the authors) to solve binary classification problems. Finally, we propose a *mapping-and-aggregation* strategy to increase the interpretability of the machine learning results. The proposed approach provides very low mis-classification errors ($<0.1$), with very few and stable MSC features ($<10\%$ of the initial set of available features). Furthermore, we identified a common pattern across algorithms and classification problems, i.e., the activation of the *centro-parietal* brain areas and *arm*’s muscles in 8-80 Hz frequency band, in line with previous literature. Thus, this study represents a step forward to the minimization of a reliable EEG-EMG setup to enable gesture recognition.

## Introduction

Modern health care is rapidly evolving from a hospital-centered service toward ubiquitous and personalized medicine. In particular, mobile and remote health care are seeing rapid developments thanks to new technologies, such as artificial intelligence (AI) and 5G [1]. Mobile health (m-health) encompasses the measurement of both vitals and physiological signals of individuals who freely move in a variety of indoor and outdoor environments, through wearables, smart and portable physiological sensors such a Inertial Measurement Unit (IMU), smart clothes, watches and electronic wrist bands. These sensing devices are increasingly employed to continuously monitor people’s health conditions, e.g., during a recovery period at home, but also for fitness purposes and advanced sport training. The m-health systems are particularly challenged to ensure stable and reliable communication links, despite the high heterogeneity of the data traffic and user needs. Also, minimal monitoring system setup is preferred to improve usability and portability and to reduce its obtrusiveness, thus facilitating the acceptability of such assistive technologies [2] and increasing the users’ Quality of Life (QoL) [3]. The above requirements were further exacerbated by the SARS-Cov2 pandemics, when the digitalization of health care and the possibility of delivering healthcare services at the patient’s home (i.e., tele-rehabilitation) emerged as key advancements to reduce the risks associated to new infections, but also to reduce the economic burden of hospitalization and travel costs for the national healthcare systems and the patients.

One of the most common applications of m-health is to provide rehabilitation and motor training at home. In this scenario, it is of utmost importance to be able to precisely recognise gestures and movements. This is typically implemented through the use of IMU sensors [4, 5], cameras [6–8] and radar sensors [9]. These kinds of monitoring can offer a high-level estimation of the movement being performed by the individual. In parallel, to explore the neural basis of the movement and to evaluate muscular fatigue as well as precise muscular control during the recovery period, electrophysiological sensing is needed [10–14]. In this paper, we focus on multi-modal systems that are able to simultaneously acquire electroencephalography (EEG) and electromyography (EMG) data during movement. Despite their higher explanatory value, these sensing modalities share a lower usability and a higher obtrusiveness: in fact, to capture significant patterns associated to motor recovery, a number of sensors are generally used to acquire brain signals from all over the scalp and from several muscles along the moving limb [15–19]. To ensure the sustainability of m-health systems based on electrophysiological measurements, one important challenge is to extract relevant pieces of EEG and EMG information to correlate with the subject’s conditions and behavior. Given the complexity of this scenario, the most common solution is to rely on expert features and previous experience, and to limit a-priori the number of sensors to a few of them, thus possibly losing relevant information related to anomalies. On the other hand, commercial low-cost EEG and EMG devices are driven by comfort to place the electrodes in convenient places (e.g., the forehead for EEG), rather than in more meaningful but inconvenient locations (e.g., the center of the scalp, or the hand). However, with the recent effort on wearables and new sensing technologies (e.g., flexible, printable and graphene-based electrodes [16, 20–22]), new solutions are expected to trade-off comfort and proper motor control monitoring.

With this perspective, we adopted an *agnostic* (rather than *expert*) and *extensive* (rather than *selective*) approach to classify hand movements using a multi-modal EEG-EMG publicly available dataset. Particularly, from the same dataset, we set up two separate classification problems to distinguish hand movements during holding of objects with different textures (i.e., the first classification problem) or weights (i.e., the second classification problem). We extracted a well-known feature, i.e., the magnitude squared coherence (MSC), from every pair of EEG and EMG sensors, and we used it for the classification. We employed three different feature selection and classification algorithms, namely feature selection with consensus (FeSC), p-value filtering with forward sequential feature selection (pFSFS), and least absolute shrinkage and selection operator (LASSO), to find the smallest subset of highly discriminative features that can effectively classify hand movements. Moreover, we studied the stability of the features of that subset and we compared the selections made by the algorithms. An individual feature is considered as *stable* when it is consistently selected across different partitions of the dataset. To this aim, we also proposed an intuitive *mapping-and-aggregation strategy* that allowed us to map all the features that were selected more often when changing the data into an aggregated frequency-EEG-EMG space that is easier to be physiologically interpreted. With this approach, we were able to provide the following contributions:

1. the systematic comparison of three different feature selection algorithms using a novel sensor-level fusion approach for the EEG and EMG signals;
2. a significant improvement of the FeSC algorithm, previously proposed in [23];
3. very high classification performance in both classification problems, comparable or higher than previous works;
4. the identification of a subset of physiologically meaningful features for the best performing classification algorithms;
5. an intuitive visual representation, via the *mapping-and-aggregation strategy*, of the subsets identified in 3 to facilitate the physiological interpretation of the results;
6. the assessment of the stability of the subsets identified in 3 across datasets, classification problems, and algorithms;
7. the identification of a predominant common pattern, corresponding to the particular activation of the *centro-parietal* brain areas and the muscles of the *arm* and *forearm* in the (8, 80) Hz frequency band, which is in line with previous literature on EEG and EMG during motor tasks.

The rest of the paper is organized as follows: Sect. 2 reports previous work that deals with data fusion in datasets including both EEG and EMG data. Also, we describe the rationale behind consensus clustering for feature selection (used in our current version of FeSC). In Sect. 3, first we explain the details of the data preparation and the MSC extraction. We describe the improved FeSC pipeline, as well as how pFSFS and LASSO algorithms work. Then, we introduce our original *mapping-and-aggregation* strategy and we define the region of interests (ROIs) used to analyze results. Finally, we present the performance metrics used to evaluate the classification outcomes from the different algorithms. Section 4 describes the dataset used in this study and provides all the results, with a critical discussion with respect to the current reference literature. Section 5 concludes our work, discussing a few limitations of this study and providing some stimulating future perspectives.

## Related works

In this section, we provide a brief review of the state of the art related to multi-modal fusion of EEG and EMG data in different scenarios. Furthermore, we also introduce previous literature on feature selection based on clustering to provide the context for the development of our original FeSC algorithm.

### EEG-EMG fusion

Feature selection is recognized as a critical step in many e-health applications, e.g., emotion recognition, anomaly detection, motor training, gesture recognition [24, 25]. It is well known that finding a small number of highly discriminative features could bring significant improvements in the classification performance [18, 26], in the explainability of the results, as well as the reduction of the required communication and storage resources in m-health scenarios.

To reduce the complexity of a multi-modal dataset, including EEG and EMG signals, two approaches are possible: manual selection based on expertise or machine learning. In the first case, a few specific EEG and EMG sensors can be selected and expert features could be extracted, while discarding all the remaining data [27]. Although being a common solution, this method requires robust priors or preliminary clinical knowledge. In the second case, machine learning approach could be adopted to provide a more general and agnostic solution, taking into account the entire available dataset. It is worth mentioning that EEG and EMG have been combined in different ways with the purpose of classification. Three main fusion methods can be used: (1) sensor level, (2) feature level, and (3) classification level [28]. However, in the analysis of EEG and EMG, the last two approaches have been mostly used. In [29], the authors have recently proposed an EEG-based fall detection system that identifies the EMG contraction onset and extracts an expert EEG feature in the 800 ms period before, to detect falls in advance. In [30, 31], the authors employ features obtained from EEG and EMG, separately, to investigate how muscular fatigue can affect hand gestures classification by combining the outputs of two classifiers based on EEG or EMG only, using different weights or a Bayesian fusion approach. The last solution results in better classification performance, even when the amplitude of the EMG signal is degraded to simulate muscular fatigue. In [30], features are independently extracted from the EEG covariance matrix and from the EMG signals. Then, the authors apply a classification based on support vector machine (SVM) to classify right- versus left-hand movements, in presence of different levels of (simulated) muscular fatigue. In [31], features are also separately obtained from EEG and EMG, and the information fusion is obtained at the decision level using a Bayesian fusion approach. In particular, the authors find that the average performance of all subjects based only on EEG is 73$\%$; on the other hand if only EMG is considered the average performance is 87$\%$. Moreover, by means of the fusion approach they find an increase to 91$\%$ of the performance. In [32], the authors review a number of studies on different hybrid BCI systems where EEG and EMG are integrated to improve the ability of exoskeletons and assistive robotics to support, or enhance, the upper limb movements. They conclude that the simultaneous, or sequential, fusion of EEG and EMG data is generally beneficial for detecting both the intention of moving and the kind of movement. The improvement is reported mainly in terms of classification accuracy, going from about $70\%$ (unimodal approach, either EEG or EMG, separately) to more than $90\%$ (multimodal approach). In [33], several hand movements have been acquired with simultaneous recording of EEG and EMG in four amputees subjects. Sequential forward selection (SFS) is employed to select the most informative EEG and EMG signals to keep, based on their contribution in terms of classification accuracy. Then, four time-domain features are extracted and fed into a linear discriminant analysis (LDA) algorithm to classify hand movements. Thanks to the fusion, the classification accuracy increased to $94.2\pm 3.2\%$ ($+14\%$ over the single modality).

In [34], EEG and EMG were fused at the feature level to classify different hand movements, obtaining an accuracy of $68.24\%$, a better results if compared with the single modality ($57.78\%$ EEG only, $61\%$ EMG only). In [35] and [36], the authors considered elbow flexion and extension movements, while varying parameters, such as the speed of motion, the load to lift, and the level of muscular fatigue. They compared two different fusion strategies for task weight classification during dynamic elbow flexion-extension motion: the single classifier (SC) used the EEG and EMG features, separately extracted, as input to a support vector machine (SVM), while the weighted average classifier (WA) combined the outputs from two signal-specific SVM classifiers. The SC approach achieved an accuracy of $80.75\%$ which was a statistically significant increase over the EEG-based classifier, but not over the EMG one. When speed was used as additional information, the authors found that WA provides the best performance: an accuracy of $83.01\%$ was achieved in the classification of movements associated with three different loads. Interestingly, the authors also proposed a preliminary feature stability study: the number of times each feature was selected from EEG and EMG was computed in different subjects, revealing some degree of robustness across them. However, the authors left a deeper analysis for further investigations.

The above-mentioned studies proved that using the features from both EEG and EMG can enhance the results of the classification. More recently, a few papers investigated the possibility of classifying gestures by fusing EEG and EMG at the data level. In [37], the authors computed the spectral power correlation (SPC) extracted from a multi-modal EEG-EMG dataset and used SVM to classify hand grasping and resting states. This method outperformed the gold standard, i.e., the common spatial patterns (CSP), with an accuracy of $90\pm 4.86\%$ (CSP achieved an accuracy of $79.75\pm 5.71\%$). In our previous works [23, 38], we introduced the possibility of classifying hand movements through a well-established, physiologically meaningful, feature, i.e., the MSC, that is obtained by taking the normalized cross-spectrum between EEG and EMG (i.e., fusion at the data level). In [38], we limited our analysis to a single EEG sensor and 5 EMG sensors and we investigated how the use of data from multiple EMG sensors, via MSC, could enhance the classification performance. In fact, we could reach a classification accuracy up to $90\%$ by using 2 to 4 EMG sensors (with some variability depending on the observation period and the motor task). In [39], the correlation between the band-limited power time-courses (CBPT) of EEG and EMG was computed from a group of healthy individuals and hemiplegic patients. The CBPT was used to control a BCI-based hand orthosis and it was calculated over a suitable time period of 1 s (which falls between 3 s and 5 s within a trial, where 3 s is the instant of cue appearance) considering (8, 12) Hz as the frequency band for the EEG and (30,50) Hz for the EMG. In addition to this, C3-FDSR, Cz-FDSR, Cz-FDSL, and C4-FDSL were considered as channel combinations. The authors found that CBPT outperforms MSC. Indeed, they found an accuracy of $92.81\pm 2.09\%$ in the healthy group and $84.53\pm 4.58\%$ in the patients group if using CBPT, and an accuracy of $72.81\pm 4.9\%$ in the healthy group and $69.53\pm 4.72\%$ in the patients group if considering MSC. Finally, in [40], a deep autoencoder architecture was proposed to extract a representation based on a limited number of discriminant features from an EEG-EMG dataset. They could classify two very well-coded emotions, dominance and arousal, reaching accuracy values in the range of 65–$80\%$. However, this study extracted a latent (i.e., transformed) representation of the input dataset, showing very high classification performance but at the same time preventing from the possibility to interpret the results in their original electrophysiological domain.

In this paper, we use MSC as a fusion method and we aim to evaluate different feature selection and classification approaches to distinguish different fine hand movements. Also, we study the stability of the features selected by different algorithms in different datasets, while providing an intuitive *mapping-and-aggregation strategy* that allows for an easier physiological interpretation. Using MSC to classify gestures has rarely been used in the literature, but it can be potentially capable of providing good classification results, with the further advantage of using a feature with a well-known physiological value, thus a straightforward clinical interpretation.

### Feature selection based on clustering

Recent works leveraged unsupervised machine learning techniques for feature selection, particularly clustering methods. Hierarchical clustering has been a common choice to progressively reduce the number of features as in [41, 42]. In the first work, the relevance of different feature combinations is studied, providing an improvement of the accuracy performance using several classifiers. In [42], instead, the authors use mutual information and the coefficient of relevancy to measure the distance between and within clusters, respectively. In [43], the authors present several methods for feature selection and describe evaluation measures that can be used to compare their performance. The accuracy is found to be in the range $70\%$–$100\%$, depending on the classification strategy and the dataset under investigation. Also, they point out that clustering-based feature selection methods could retain irrelevant features: indeed, these are often clustered together and then represented by some elements in the final set of selected features.

As a solution, [44] applies clustering with consensus to identify more reliable and robust ROIs from a neuroimaging set of signals. To this aim, the authors run multiple iterations of the k-means algorithm to clusterize together multiple samples corresponding to different brain regions. Brain regions are then aggregated when consensus across iterations is found.

In one of our previous works [23], we considered 32 EEG and 5 EMG sensors, we extracted the MSC from every multi-modal pair and we used its values to form a high-dimensional feature vector that can be used to classify different hand movements. The high number of available MSC features was reduced through an original feature selection strategy based on consensus clustering, namely *FeSC* [23]. However, in its first prototype, FeSC achieved only limited classification results (accuracy around $60\%$). Therefore, here, we highly improve its performance, including a more refined pre-processing procedure and a more robust cross-validation strategy.

As far as the authors know, this particular investigation has not been proposed yet, in previous literature.

## Methods

In this section, we describe the signal processing, from its pre-processing to the extraction of the feature matrix. Then, we describe the feature selection methods we compare, namely FeSC, pFSFS, and LASSO, and define the metrics used in the evaluation of the classification performance. These algorithms have been chosen as prominent representatives of three main categories of feature selection algorithms: namely, wrapper, filtering, and embedded methods, respectively [45, 46]. Wrapper methods, such as FeSC, always determine the best feature subset based on classification; filtering methods, such as LASSO, are independent from classification, while the embedded methods, as pFSFS, combine the previous ones. The stability of the selected features has been also studied, with advantage toward the interpretability of the outcomes of the algorithms.

### Pre-processing and feature extraction

We deal with datasets including EEG and EMG signals, simultaneously acquired from a number of participants, while performing two different upper limb movements (i.e., gesture 1 and gesture 2). Moreover, we assume to have artefacts-free datasets, thus, we can perform a lightweight pre-processing, as described in the following. We apply a bandpass filter (i.e., Chebyshev type I, order 86) to every signal, regardless of their kind, to limit them to the frequency band (1.5, 80) Hz [27]. Then, we apply a notch filter (i.e., IIR filter, direct-form II, order 2) to remove the power supply interference. For each repetition of the movement, and for every EMG signal, we identify the movement onset (i.e., the time instant when the muscle begins to contract) and the activation period (i.e., the period of time where the muscle is stably contracted), by means of expert labels included in the dataset. Then, we segment the signals into 4 s-long segments, each corresponding to a single repetition of the movement. Every EEG signal is segmented according to the EMG activation. Here, it is important to note that, given that every EMG signal could have a slightly different movement onset and activation period, the EEG dataset is segmented according to each of the EMG signals: thus, we obtain multiple EEG segmented datasets, one per each available EMG signal. This is important for a proper feature extraction, as explained in the following. Finally, the EMG segments are full-wave rectified, and both EEG and EMG are normalized over their own area under curve (AUC) value.

We extract a single kind of feature, the MSC. It can be interpreted as the fusion, at the sensor level, of both the information from the EEG and the EMG [28]. Also, it has a very well-known physiological meaning associated to it, which has been well investigated in the literature related to motor control [13, 14, 47–51].

Formally, the MSC is computed as the normalized cross-spectrum between an EEG and an EMG segment. In what follows, we report the mathematical derivation of this feature.

Given *N* repetitions of the gesture to investigate, let $s_{m}^{n}(t)$ represent the *n*-th element with signal length *T* seconds, $t\in [0, T]$, and $m=1, 2, \dots , M$ the number of EMG sensors considered. Also, let $s_{q}^{n}(t)$ be the analogue signal for the EEG dataset, with $q=1, 2, \dots , Q$ the number of available EMG sensors.

Let $S_{q}^{n}(f)$ and $S_{m}^{n}(f)$ be the autospectra of the *n*-th segment of EEG and EMG, respectively, and $S_{m,q}^{n}(f)$ the EEG-EMG cross-power spectrum of the *n*-th segment. We use the Fast Fourier Transform (FFT) algorithm to compute the spectra of segments (0.5 s-long Hann window, 256 FFT points). Then, we define $S_{q}(f)$, $S_{m}(f)$, and $S_{m,q}(f)$ as the averages along the *N* segments, obtained from $S_{q}^{n}(f)$, $S_{m}^{n}(f)$, and $S_{m,q}^{n}(f)$, respectively. For every segment $n = 1, 2,..., N$, the MSC feature is computed as follows [52]:

1 $$\begin{aligned} C_{m,q}^n(f) = \dfrac{|S_{m,q}^n(f)|^2}{|S_{q}^n(f)||S_{m}^n(f)|}. \end{aligned}$$

Based on the Cauchy-Schwarz inequality, it holds

2 $$\begin{aligned} 0 \le |S_{m,q}^n(f)|^2 \le |S_{q}^n(f)||S_{m}^n(f)|. \end{aligned}$$

Thus, the $C_{m,q}^n(f)$ values range between 0 (i.e., uncorrelated signals) and 1 (i.e., perfect linear relationship). The custom Matlab implementation of the MSC computation is available on GitHub.^1^

Finally, for every segment, given $C_{m,q}^n(f)$, we extract the average MSC value from 11 well-known frequency bands defined as $\delta =(1.5, 4)$ Hz, $\theta =(4, 8$) Hz, $\alpha =(8, 13)$ Hz, $\beta _1=(13, 20)$ Hz, $\beta _2=(20, 30)$ Hz, $\varvec{\beta} =(13, 30)$ Hz, $\gamma _1=(30, 45)$ Hz, $\gamma _2=(45, 60)$ Hz, $\gamma _3=(60, 80)$ Hz, $\gamma =(30, 80)$ Hz, and the *full* band $= (1.5, 80)$ Hz. Note that some of them are overlapping with each other, e.g., $\beta _1$ and $\varvec{\beta}$, whereas others cover multiple narrower sub-bands, e.g., $\varvec{\beta}$ includes $\beta _1$ and $\beta _2$. However, they can be useful as they are often considered in neuroscience as $\beta _{low}$ and $\beta _{high}$ for their different role in the brain processes [53].

Then, for every movement repetition *n* and for each class *c*, we can define the feature vector, ${\mathbf {x}}_{\mathbf {c}}$, as the set of average MSC values in all available combinations of EEG, EMG, and frequency band as follows (the bar sign corresponds to the average over the frequency bins of each specific frequency band)

3 $$\begin{aligned} \begin{aligned} {\mathbf {x}}_{\mathbf {c}}(n) = [ \overline{C_{1,1}^n(\delta )}, \overline{C_{1,1}^n(\theta )},..., \overline{C_{1,1}^n(\mathrm full)}, \overline{C_{1,2}^n(\delta )}, \overline{C_{1,2}^n(\theta )},..., \overline{C_{M,Q}^n(\mathrm full)}]. \end{aligned} \end{aligned}$$

Therefore, ${\mathbf {x}}_{\mathbf {c}}$ contains $M \times Q \times K$ MSC samples, with *M* and *Q* being the number of EMG and EEG available sensors, respectively, and $K=11$.

It is worth noting that this step typically produces a high number of extracted features. Therefore, here the need for an automatic procedure to select a limited number of relevant features comes into play, in order to improve the subsequent classification and to decrease the computational effort.

Finally, we form the feature matrix, ${\mathbf {S}}$:

4 $$\begin{aligned} \begin{aligned} {\mathbf {S}} = [&{{\textbf {x}}}_{{\textbf {c1}}}(1), \mathbf {x_{c1}}(2), \ldots , \mathbf {x_{c1}}(N_1), \mathbf {x_{c1}}(N_1+1), \ldots , \mathbf {x_{c1}}(N_1+N_2), \\&\mathbf {x_{c2}}(1), \mathbf {x_{c2}}(2), \ldots , \mathbf {x_{c2}}(N_1), \mathbf {x_{c2}}(N_1+1), \ldots , \mathbf {x_{c2}}(N_1+N_2) ]^T, \end{aligned} \end{aligned}$$

where $N_1$ and $N_2$ represent the samples corresponding to all the $N_1 + N_2 = N$ repetitions of the gestures, and *c*1, *c*2 are the two classes that we consider in the classification problem.

Given that the datasets used in this work include an imbalanced number of segments both across subjects and gestures, we apply Synthetic Minority Over-sampling Technique (SMOTE) [54] at the subject level. SMOTE consists in augmenting the dataset, using the available data (where each sample is represented by one feature vector) and adding one or more equally spaced samples, depending on the desired oversampling factor, in between each pair of samples. For this step, we first perform a normalization within-subject (over the maximum). Then, we apply SMOTE to balance the number of segments between gestures in each single subject.

### Feature selection algorithms

In this section, we introduce the feature selection algorithms that we compare in the following discussion, namely FeSC, pFSFS, and LASSO. To evaluate the classification performance we partitioned data to realize a repeated ($R=10$) holdout cross-validation (CV) procedure, where $90\%$ of the feature matrix ${\mathbf {S}}$ obtained from the pre-processed data is used to train the algorithm, and the remaining $10\%$ is kept for the test phase, where the performance of the trained model is evaluated over unseen data.

#### FeSC

First presented in [23], Feature selection with consensus (FeSC) is based on machine learning and aims to robustly select the most relevant MSC features to classify different fine hand movements. In the version used in this paper, FeSC has been significantly revised and improved and now it includes 3 clustering algorithms. FeSC has been implemented in Matlab using custom code.^2^

FeSC is made by three main steps: pooling, consensus clustering, and classification, better described in the following.

*Pooling* As the first step, FeSC performs a pooling of the dataset to reduce in a two-dimensional space the elements that characterize each feature: specifically, it takes the average of MSC across all trials of the same class. Then, the resulting matrix is transposed. The feature matrix ${\mathbf {S}}$ described in Eq. (4) thus results in the reduced matrix $\mathbf {S_r}$, defined as

5 $$\begin{aligned} \mathbf {S_r} = \left[ \overline{\mathbf {x_{c1}}}, \quad \overline{\mathbf {x_{c2}}} \right] ^T. \end{aligned}$$

*Consensus clustering* The second step consists in the implementation of a consensus clustering algorithm among three different clustering algorithms, similar to the approach applied in [44]. The evaluation of the consensus makes it possible to identify which features are grouped together by more than one individual clustering algorithm and, hence, ensures that the final clusterization is less affected by the specific choices of the clustering algorithm.

The consensus clustering algorithm takes ${\mathbf {S}}_r^T$ as input and clusterizes the available features into the $M_r$ clusters that collect the largest consensus among the different clustering algorithms. The consensus clustering algorithm mainly depends on two parameters: the similarity index, $\sigma$, which is the fraction of individual clustering algorithms that agree on a certain feature selection; and $\nu$, the minimum average size of the clusters. During the consensus procedure, therefore, groups of more than $\nu$ features that have mutual similarity index larger than $\sigma$ are clustered together. While $\nu$ is learned during the training of FeSC, as reported in [23], we set $\sigma$ either to 0.6, which allows for partial agreement among the clustering algorithms (at least two out of three) to accept a feature in a certain cluster, or to 0.9 when we want to force full agreement among the clustering algorithms. Then, the centroid of each cluster, i.e., one representative feature from each of them, is selected.

In this work, we consider the hierarchical clustering, the spectral clustering, and the Density-Based Spatial Clustering of Applications with Noise (DBSCAN) as individual clustering algorithms for the consensus.

In the following, the combination of algorithms used in the consensus step will be identified by the initial letter of the algorithm: (H) hierarchical, (S) spectral, (D) DBSCAN clustering.

*Classification* Finally, FeSC implements a kernel-SVM classifier [55]. Based on our previous study [23], we decided to use the radial basis function (rbf) kernel, as it outperforms the classification with respect to other kernels. Note that the selection of features may depend on the specific data considered in the fold. Thus, the classification performance achieved in this step is employed to identify the selection of the $M_r$ most discriminative features to maintain. To optimize the FeSC parameters, we applied a nested 5-fold CV procedure, selecting the combination of features that minimized the mean classification error (MCE).

#### pFSFS

This method makes use of a sequential feature selection algorithm to select the most representative features to classify different gestures, and it was modified from [56].

pFSFS is a 3-step recursive procedure including: (i) the application of a filter based on the p-value, (ii) the application of the sequential feature selection, and (iii) the training of a quadratic discriminant analysis (QDA) model.

The* p*-value gives the probability that the samples of the first class belong to the same statistical distribution as the samples of the second class. Then, first, filtering based on p-value is operated on the feature matrix to remove those features, i.e., columns, which have a p-value above a pre-determined threshold (typically, 0.05). Second, the forward sequential feature selection (F-SFS) algorithm is used in a wrapper fashion to obtain a ranked selection of the most relevant features to explain the difference between the two classes. Third, a QDA classifier is employed to classify the dataset based on the selected features. As a loss function for parameter optimization, we used the MCE.

This method was implemented in Matlab, making use of the *sequentialfs.m* library function and other custom code.

#### LASSO

LASSO is a well-known linear regression method aiming at selecting the most discriminative features (i.e., the predictor variables) to explain the class samples (i.e., the responses), taking into account the cross-correlation between features [57, 58]. LASSO performs an L1-regularization by minimizing the sum of the squared errors provided by the model (i.e., as in the ordinary least squares objective function) with a penalty given by the weighted sum of the model’s coefficients (i.e., the vector $\varvec{\beta}$). Such weights, defined by the hyperparameter $\uplambda$ (i.e., the LASSO sparseness parameter), determine the regularization that is performed by the procedure on the model. For any given nonnegative $\uplambda$ value, the LASSO objective function is expressed by

6 $$\begin{aligned} {\mathscr {L}} = \frac{1}{2N} \sum _{i=1}^N(y_i - \beta _0 + {\textbf {x}}_{\textbf {i}}^{\textbf {T}}{\varvec{{\beta }}})^2 + \uplambda \sum _{j=1}^p |\beta _j|, \end{aligned}$$

where *N* is the number of samples, ${{\textbf {x}}}_{{\textbf {i}}}$ is the *i*-th sample of length *p*, $y_i$ is the true class of ${{\textbf {x}}}_{{\textbf {i}}}$, $\beta _0$ is a scalar, and $\beta =[\beta _1,..., \beta _p]$ is the vector of model’s coefficients. The choice of $\uplambda$ is particularly critical and it is typically learnt from the data through cross-validation [58]. Here, in order to properly choose $\uplambda$ and the coefficients $\varvec{\beta}$, we performed a 5-fold CV (to be fair, we used the same partitions as FeSC) and we selected their values according to the model with the minimum mean square error (MSE). Matlab’s native grid-search parameter optimization was exploited. Features were ranked by decreasing values of the $\varvec{\beta}$ coefficients in the LASSO model. Those with zero-valued coefficients were discarded. Then, we trained an SVM with an increasing number of relevant features, starting from the one with the largest $\varvec{\beta}$ coefficient, and used the MCE as a loss function for SVM parameter optimization.

This method was implemented in Matlab, making use of the *lasso.m* library function and other custom code.

### Features stability and interpretability

We also investigated the stability of the selected features across different subsets of the same dataset. As mentioned before, we obtained *R* different splits of the dataset during the CV procedure (see Sect. 3.2) and run the feature selection with classification for each of them. Finally, the results were compared across repetitions to evaluate the stability of the selected features. To quantify stability, for each algorithm, we retained those features that were selected in at least $70\%$ of the repetitions. We were able to find a set of stable features for each algorithm and for each classification problem.

In order to facilitate the comparison of the results among different algorithms, and to enhance the interpretability of the results, we also developed a *mapping-and-aggregation* strategy that works as follows. First, every stable feature was mapped back to its original EMG, EEG, and frequency domains. This is possible because every MSC feature was obtained by a specific combination of EMG and EEG sensors and frequency band, thus providing an invertible map. Second, we selected a few suitable ROIs, in each domain, to aggregate features. Particularly, the frequency bands were aggregated into 3 ROIs, namely *low frequencies*, *sensorimotor rhythms (SMR)* and *full*. *Low frequencies* range includes the $\delta$ and the $\theta$ bands; the *sensorimotor rhythms* has a well-known neurophysiological meaning and its range includes the bands $\alpha$, $\beta _1$, $\beta _2$, $\varvec{\beta}$, $\gamma _1$, $\gamma _2$, $\gamma _3$, and $\gamma$, while *full* considers the entire frequency spectrum taken as a whole (i.e., it includes only the full band). For the aggregation of the EEG sensors, we slightly modified the approach of [59]: while in [59] the authors identified six ROIs, here we only have three of them: the *Frontal*, the *Centro-parietal*, and the *Occipital* one. Specifically, we included the original temporal, central and parietal EEG ROIs into the new *Centro-parietal* ROI. This is motivated by the fact that the reference article dealt with cognitive processes, while our study investigates motor-related brain activity. From well-consolidated literature on motor control, we know that several brain areas are involved in the control of the hand movements (i.e., the primary motor cortex, the posterior parietal cortex, the premotor cortex, and the supplementary motor cortex) [60]. They include all EEG sensors located at the central, parietal and temporal areas; thus, we decided to have them in one single ROI. Finally, the EMG sensors have been aggregated to distinguish between *Arm*, *Forearm*, and *Hand*. In *Arm*, we included AD and BR; in *Forearm*, we included CED and FD; in *Hand* region, we only have FDI.

### Performance evaluation

To evaluate the classification performance of each algorithm, we computed the confusion matrix and the most common classification metrics, including the MCE, the True Positive Rate (TPR), the True Negative Rate (TNR), and the F1 score (F1-score) over the test set (at each repetition). They are computed as follows:

7 $$\begin{aligned}{\rm MCE}&= \frac{ \rm {FP}+{\rm FN}}{{\rm TP}+{\rm TN}+{\rm FP}+{\rm FN}}; \end{aligned}$$

8 $$\begin{aligned} {\rm TPR}&= \frac{\rm TP}{\rm {TP}+{\rm FN}} ; \end{aligned}$$

9 $$\begin{aligned} {\rm TNR}&= \frac{\rm TN}{{\rm TN}+{\rm FP}} ; \end{aligned}$$

10 $$\begin{aligned} F1&= \frac{2}{2{\rm TP}+{\rm FP}+{\rm FN}}. \end{aligned}$$

where TP, TN, FP, and FN represent true positives, true negatives, false positives and false negatives, respectively.

Particularly, we analyzed the F1-score to compare different models.

## Results and discussion

We investigated FeSC, pFSFS, and LASSO over two EEG-EMG datasets. For FeSC, we considered all possible combinations of three clustering algorithms in the consensus block, namely FeSC-HS, FeSC-HD, FeSC-SD (we remind that H stands for Hierarchical clustering, S for spectral clustering, and D for DBSCAN clustering). Also, FeSC was evaluated using three clustering algorithms, and two settings, namely $\sigma =0.6$ and $\sigma =0.9$. These versions of FeSC are indicated as FeSC-HSD. Here, we describe the dataset and report the most significant results.

### WAY-EEG-GAL dataset

We considered the publicly-available WAY-EEG-GAL dataset [61], where EEG and EMG data were simultaneously obtained while participants were performing repetitions of a grasp-and-lift task. For each repetition, they had to grasp an object with their right thumb and index fingers, lift it up to an a-priori selected position, hold it for a few seconds, release the object, and return to the initial position. At each repetition, the object was randomly changed in weight, surface friction or both. From this dataset, we extracted two datasets that allowed us to study two different classification problems: (i) the sandpaper-silk (SS) problem, aiming at classifying hand movements when the objects have different surface frictions (i.e., sandpaper, as *class 1* or silk, as *class 2*), or (ii) the light-heavy (LH) problem, aiming at classifying hand movements when the objects have different weights (i.e., light, as *class 1*, and heavy, as *class 2*). Thus, we obtained the SS dataset and the LH dataset. The acquisition setup consisted in 10 EMG sensors, placed over 5 different muscles of the right upper limb acquiring 5 bipolar EMG signals ($M=5$), and 32 EEG sensors ($Q=32$) placed at standard locations according to the International 10-20 EEG System [62].

**Table 1:** Number of EEG and EMG segments available in each class of each classification problem

Subject Id.	SS		LH	
	Class 1 (sandpaper)	Class 2 (silk)	Class 1 (light)	Class 2 (heavy)
P1	51	220	84	57
P4	39	210	84	57
P7	51	221	84	57
P11	50	221	84	57
P2	50	221	84	57
P3	51	220	84	84
P5	50	221	84	30
P9	51	220	84	57
Total	393	1754	672	456

First, we downsampled the EMG signals to 500 Hz, in order to have the same sampling frequency as the EEG. The bit resolution was 12 bit for both kinds of signals. We extracted data from 8 subjects (excluding subjects and sessions where experimental records annotated any acquisition problems). Table 1 reports the number of segments available for each subject, for each class of gestures. Note that the same number of segments has been extracted from the dataset both for the EEG and the EMG. Also, for the sake of clarity, note that in this paper we identify one data *segment* with one classification *sample*.

We noticed that classes were highly imbalanced (ratio 4 : 1) in the SS dataset and slightly imbalanced (ratio 2 : 1) in the LH dataset, then we decided to apply over-sampling via SMOTE [54]. Thus, we obtained 1768 samples for each class of the SS dataset, and 672 samples for each class of the LH dataset.

Finally, we extracted the average MSC values in $K=11$ frequency bands of interest, which gave us a total number of features equal to $5 \times 32 \times 11 = 1760$ in the feature matrix ${\mathbf {S}}$, for each repetition of the movement, from each dataset.

### Classification results

First, we evaluated the classification performance over the training set in terms of MCE, as well as in 10 different, unseen, test sets in terms of MCE, TPR, TNR, and F1-score, for all algorithms and configurations. Tables 2 and 3 report the related results.

**Table 2:** Training and test MCE in the SS dataset across 10 runs. Results are expressed in terms of average (standard deviation)

Model	Training MCE	Test MCE	TPR	TNR	F1
FeSC-HS	0.0089 (0.0006)	0.0071 (0.0049)	0.9163 (0.2880)	0.9051 (0.2819)	0.9106 (0.2853)
FeSC-SD	0.0116 (0.0008)	0.0147 (0.0081)	0.9966 (0.0072)	0.9739 (0.0145)	0.9855 (0.0079)
FeSC-HD	0.0121 (0.0006)	0.0133 (0.0065)	0.9966 (0.0072)	0.9768 (0.0102)	0.9868 (0.0065)
FeSC-HSD-0.6	0.0107 (0.0008)	0.0110 (0.0052)	0.9966 (0.0061)	0.9813 (0.0097)	0.9891 (0.0052)
FeSC-HSD-0.9	0.0109 (0.0008)	0.0082 (0.0056)	0.8949 (0.3145)	0.8904 (0.3131)	0.8927 (0.3137)
pFSFS	0.0007 (0.0005)	0.0048 (0.0048)	0.9932 (0.0084)	0.9972 (0.0040)	0.9952 (0.0049)
LASSO	0 (0)	0.0110 (0.0047)	0.9909 ( 0.0061)	0.9909 (0.0061)	0.6646 (0.0014)

Precision, recall, and F1-score are also included

**Table 3:** Training and test MCE in the LH dataset across 10 runs. Results are expressed in terms of average (standard deviation)

Model	Training	Test	TPR	TNR	F1
FeSC-HS	0.1320 (0.0040)	0.1127 (0.0331)	0.8279 (0.2517)	0.8049 (0.2439)	0.8174 (0.2489)
FeSC-SD	0.1742 (0.0047)	0.1799 (0.0368)	0.8254 (0.0483)	0.8149 (0.0387)	0.8208 (0.0375)
FeSC-HD	0.1823 (0.0040)	0.1537 (0.0315)	0.8433 (0.0412)	0.8493 (0.0382)	0.8457 (0.0324)
FeSC-HSD-0.6	0.1717 (0.0049)	0.1552 (0.0283)	0.8299 (0.0452)	0.8597 (0.0346)	0.8421 (0.0301)
FeSC-HSD-0.9	0.1757 (0.0044)	0.1642 (0.0382)	0.6797 (0.3515)	0.6725 (0.3478)	0.6765 (0.3497)
pFSFS	0.0308 (0.0089)	0.0604 (0.0181)	0.9090 (0.0355)	0.9701 (0.0157)	0.9374 (0.0196)
LASSO	0(0)	0.2007 (0.0308)	0.6940 (0.0577)	0.6940 (0.0577)	0.5804 (0.0205)

Precision, recall, and F1-score are also included

We show that, regardless to the model used, we could reach very low MCE values in both classification problems using the MSC features: particularly, the test MCE is always lower than 0.015, and 0.2 for the SS and LH problems, respectively. Also, we could obtain very high F1-score values for most of the algorithms: in the SS problem, except for LASSO, the other algorithms achieve an F1-score higher than 0.89. In the LH problem, both LASSO and FeSC-HSD-0.9 under-perform, while the others reached F1-score values above 0.82.

We observed that, in general, we could get better performance on the SS dataset, compared to the LH, irrespective of the algorithm used: this could be due either to the higher size of the first dataset (2.5 times larger than the other), or to the higher significance of the MSC feature for the SS dataset. Finally, we have to highlight that some models performed better than others in terms of standard deviation: FeSC-HS and FeSC-HSD-0.9 showed large standard deviations (exceeding 0.2) in the classification metrics of both the SS dataset and the LH dataset. On the other hand, the other models showed much smaller values (around 0.01 or less).

Thus, we focus on the models with high F1-score and smaller standard deviations which are more reliable. Hence, in the following, we present further results limitedly to FeSC-SD, FeSC-HD, FeSC-HSD-0.6, pFSFS.

### Feature selection and stability

**Table 4:** Selected and stable features in the SS dataset

**Model**	Mean value (Standard deviation)	**Stable features [no.]**	**Selection efficiency** [$\%$]
FeSC-HD	183 (13.2)	1	$0.5\%$
FeSC-HSD-0.6	188 (8.2)	1	$0.5\%$
FeSC-SD	182 (11.6)	3	$1.6\%$
pFSFS	79.4 (9.6)	25	$31.5\%$

Now on, we limit our analysis to robust models and investigate another interesting aspect: the ability of the algorithms to extract few, very informative, features among the 1760 available ones. This represents a preliminary, yet critical, step toward the future minimization of the acquisition setup for mobile health applications. Tables 4 and 5 provide a detailed analysis of the selected features that provided the minimum MCE value (across the test sets): the second column reports the average number of features across test sets (and its standard deviation); the third column shows the number of stable features among them; the last column reports the *selection efficiency*, an index we introduced to quantify the percentage of stable features over the average number of selected features. The higher the selection efficiency, the more robust the feature selection to dataset variations (i.e., different folds of the same dataset). It is worth noting that there is a correspondence between Tables 2 and 4 and between Tables 3 and 5. For example, the performance shown in the third row of Table 2, related to the FeSC-HD model, has been obtained considering the 183 selected features mentioned in the first row of Table 4.

**Table 5:** Selected and stable features in the LH dataset

**Model**	Mean value (Standard deviation)	**Stable features [no.]**	**Selection efficiency** [$\%$]
FeSC-HSD-0.6	181 (8.4)	1	$0.5\%$
FeSC-HD	189 (10.7)	2	$1.06\%$
FeSC-SD	191 (8.4)	3	$1\%$
pFSFS	95.3 (10.5)	45	$47.2\%$

Based on the results, we can claim that all models are able to identify a very small subset of features (between 80 and 200, representing about $4\div 10\%$ over the total number of available features) that can lead to an optimal classification. This is also consistent across different models and different datasets.

Moreover, we can observe that FeSC models are able to identify a very limited number of stable features. On the other hand, pFSFS seems to be the best model in terms of selection efficiency: it selects a lower number of features to reach minimum MCE and at least $30\%$ of them are stable. This is probably due to the initial filtering step that is not present in FeSC methods.

Therefore, we can conclude that a trade-off exists between the number of stable features that an algorithm can extract and the selection efficiency that it can achieve. The algorithm which corresponds to the best choice finally depends on the specific constraints of the application: if no strict limitations are required over the number of features (i.e., sensors) to use, then pFSFS is the best choice. On the other hand, if only a few features can be selected, then FeSC represents the best option. We stress out that we did not force the algorithms to choose a limited number of features, but some of them (i.e., the FeSC variants) can help us more to identify fewer stable features without a-priori filtering (as in pFSFS), while achieving similar high classification performance.

**Fig. 1:**
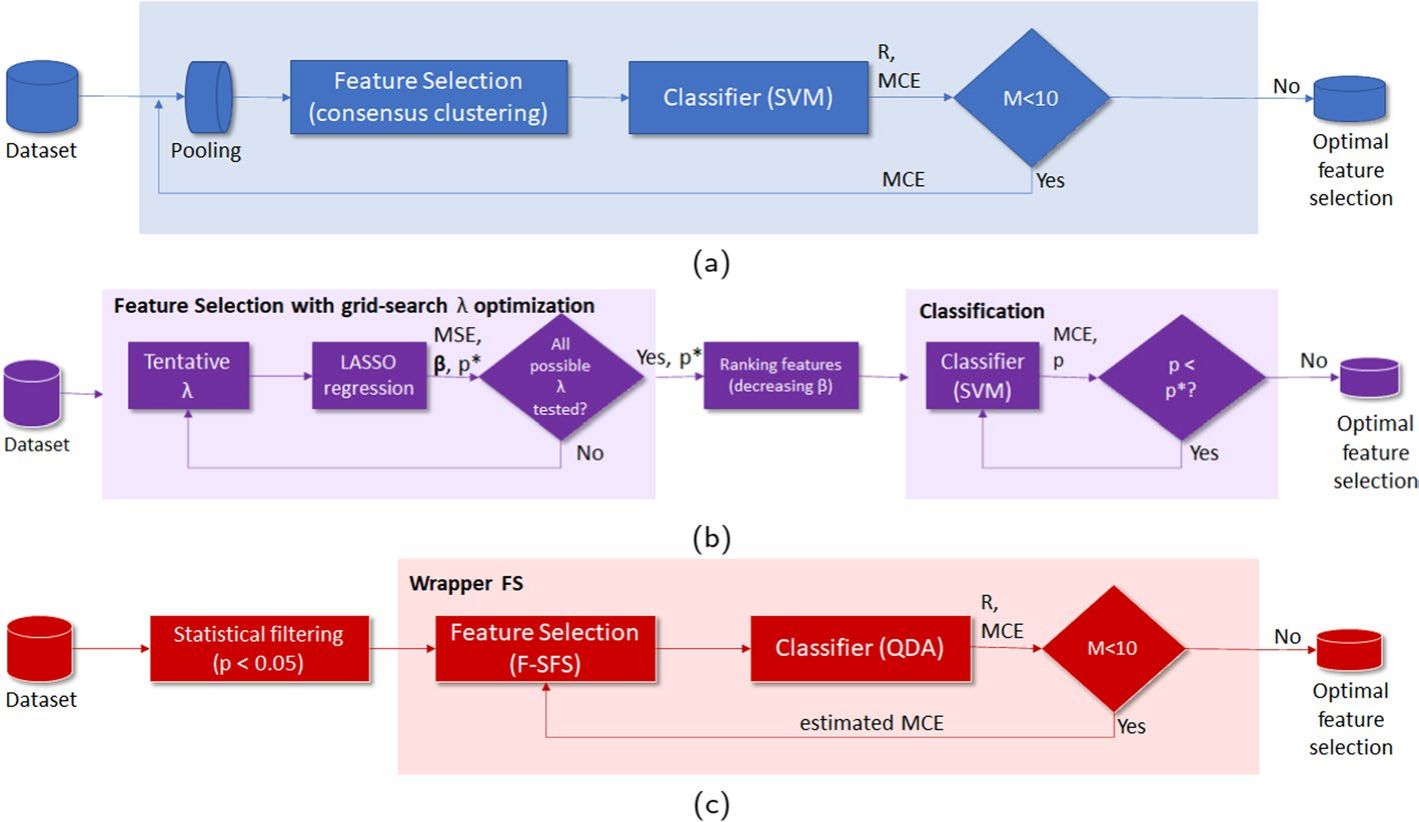
Block schemes for the three feature selection algorithms: (a) FeSC, (b) LASSO, (c) pFSFS

Finally, it is worth noting that pFSFS (with QDA classifier) is the fastest algorithm to run but, at the same time, it might not be able to effectively handle outliers in the dataset [63], as much as FeSC that implements an SVM. This results in a more general applicability and flexibility of FeSC, compared to pFSFS (Fig. 1).

**Fig. 2:**
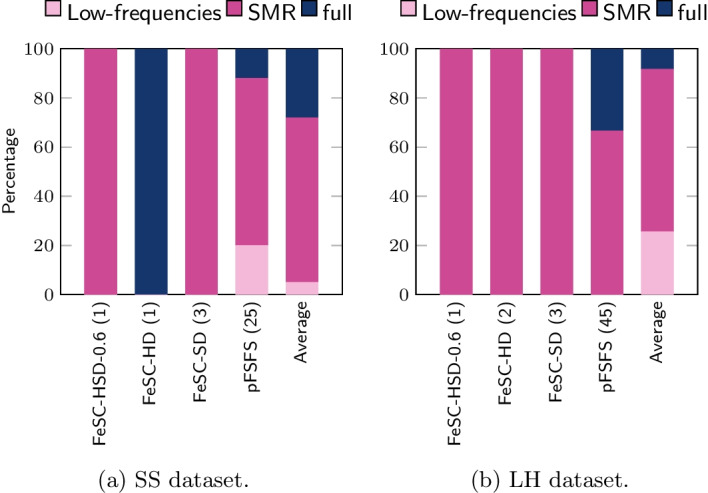
Percentage of iterations in which each frequency feature was selected as stable by the different algorithms (threshold was set to 70%). To note, algorithms were sorted by the increasing number of stable features

**Fig. 3:**
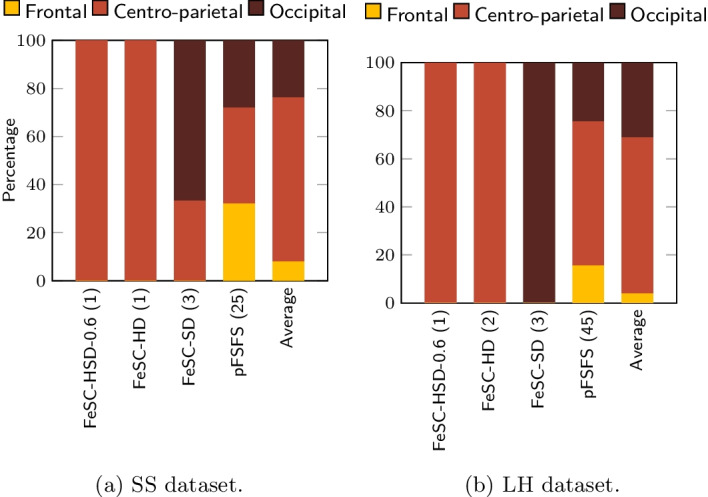
Percentage of iterations in which each EEG feature was selected as stable by the different algorithms (threshold was set to 70%). To note, algorithms were sorted by the increasing number of stable features

**Fig. 4:**
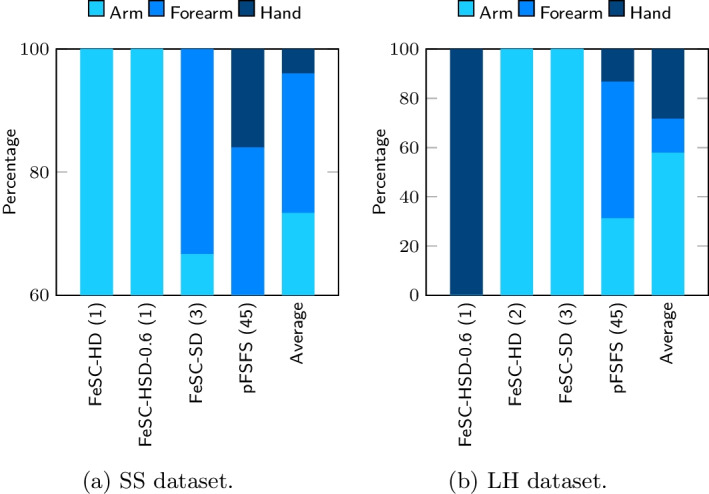
Percentage of iterations in which each EMG feature was selected as stable by the different algorithms (threshold was set to 70%). To note, algorithms were sorted by the increasing number of stable features

Figures 2, 3, 4 show the results of the mapping-and-aggregation for frequency bands, EEG and EMG sensors, respectively. This procedure was operated on the SS and LH datasets, separately. First, we consider the number of stable features identified by each method; then, we map them back into their original domains, i.e., frequency bands, EEG and EMG sensors, and aggregate them into physiologically meaningful ROI, to further facilitate their interpretation (see Sect. 3.3).

Even though we found only a limited number of stable features, a common pattern is observed in all methods, across different datasets: as for the frequency bands selection, the average percentage of SMR features ($67\%$ for SS, $66.1\%$ for the LH) is much higher compared to the lower frequencies ($5\%$ for SS, $25.6\%$ for the LH) and the *Full* band ($28\%$ for SS, $8.3\%$ for the LH). In the selection of the EMG ROI, the involvement of the arm ($73.33\%$ for SS, $57.8\%$ for the LH) is larger than that of the forearm ($22.67\%$ for SS, $13.9\%$ for the LH) and the hand ($4\%$ for SS, $28.3\%$ for the LH). Finally, for the EEG domain, we observe that the most involved stable features lay over the *centro-parietal* ROI ($68.3\%$ for SS, $64.99\%$ for the LH), then on the *occipital* ROI ($23.7\%$ for SS, $31.1\%$ for the LH), and minimally on the *frontal* ROI ($8\%$ for SS, $3.9\%$ for the LH).

Overall, the *SMR*, *centro-parietal*, *arm* pattern emerges in all methods, across the datasets and this is in line with well-established medical literature [48, 50, 64, 65].

Unexpectedly, we could not find any significant difference in patterns between the two different datasets (i.e., corresponding to different motor tasks), nor we could identify more detailed patterns, beyond the ROIs. However, we might speculate that one reason is that the two datasets are not completely separated datasets. In fact, in the WAY-EEG-GAL dataset, the objects always have two properties: a weight property (light, heavy) and a texture property (sandpaper, silk). In this paper, we set up two classification problems to distinguish, one at the time, different textures or different weights. The features that came out from the feature selection procedure are those that maximally discriminate, one at the time, the weight or the texture. Further investigations on the physiological interpretation of the results were left beyond the scope of this paper.

## Conclusions and future perspectives

In this work, we adopted an agnostic and extensive approach to classify hand gestures from a set of multi-channel EEG and EMG sensors, with the objective of making a step toward the minimization of the setup for m-health applications (e.g., in-home rehabilitation), while ensuring the highest classification performance.

The MSC, well-known physiologically meaningful feature, has been computed from any available pair of EEG and EMG sensors, in 11 common frequency bands of interest. The new high-dimensional input representation (i.e., with 1760 MSC features) motivated the use of feature selection algorithms, which have been compared considering the performance in the classification of hand gestures. In particular, we considered pFSFS, several variations of FeSC and LASSO. Note that the robustness and effectiveness of FeSC, previously proposed in [23], have been highly enhanced in this study. We studied the stability of selected features across different data partitions and two different datasets. To facilitate the interpretation of the results, we proposed a brand-new intuitive aggregation-and-mapping strategy that allows to map all stable features into an aggregated EMG-EEG-frequency space, with physiologically meaningful ROIs. Also, we introduced the selection efficiency index which allows to evaluate the robustness of the feature selection across different subsets.

Our results show that, regardless of the model used, we could reach very accurate classification performance in both datasets, using the MSC features. We then compared the best models (i.e., those with highest F1-score and lowest standard deviation values in the classification metrics) as for their ability to extract few and stable features. These models are able to identify a very small subset of stable features (between 1 and 45, representing about $0.06\%\div 2.7\%$ of the total number of available features) ensuring, at the same time, optimal classification. This result was consistent across different datasets. Also, we highlighted the advantages and disadvantages of selecting each specific feature selection algorithm: overall, we found out that pFSFS is more effective and fast in the selection of the subset of features to represent the entire dataset, compared to FeSC. At the same time, FeSC can identify a few, very informative features without using the p-value-based filter, which assumes the data to be normally distributed.

Furthermore, with the mapping-and-aggregation strategy it was possible to identify a common pattern, namely the prominent activation of *centro-parietal* brain areas and the muscles of the *arm* in the 8-80 Hz frequency band, across different methods and datasets, in line with previous literature on EEG and EMG during motor tasks.

This work still presents some limitations. The computational efficiency of FeSC can be increased by selecting faster individual clustering algorithms (e.g., DBSCAN was much faster than spectral clustering on our development platform). Furthermore, an extended study on the stability of the features could be performed. For example, it could be interesting to investigate their stability not only across several partitions of each dataset, but also across different subjects. This may reveal subject-specific patterns that may vary stable feature selection to some extent. Finally, the conclusions of our work could be further tested in other datasets with different motor tasks (e.g., other arm movements, lower limb movements, gait [19]), or in different contexts, e.g., emotion recognition and anomaly detection, where multi-modal datasets are available. Additionally, a comparison between different data fusion strategies (e.g., feature-level fusion) is needed, to determine whether the data-level fusion proposed here (using MSC) is more effective than other, more common, approaches [17, 28, 29].

This study and its further developments represents a step forward to enable a portable technology to support motor training at home, through wearables, in a near future. To this aim, minimizing the acquisition setup while, at the same time, ensuring high performance of gesture recognition is the key to promote m-health and to provide reliable and usable healthcare systems closer to the patients.

## Data Availability

The datasets generated and/or analyzed during the current study are available at https://figshare.com/collections/WAY_EEG_GAL_Multi_channel_EEG_Recordings_During_3_936_Grasp_and_Lift_Trials_with_Varying_Weight_and_Friction/988376.

## Footnotes

1 The code is available at https://github.com/CisottoGiulia/EEG-EMG-analytics.

2 The code is available at https://signetlabdei.github.io/FeSC
